# The crosstalk between FGF21 and GH leads to weakened GH receptor signaling and IGF1 expression and is associated with growth failure in very preterm infants

**DOI:** 10.3389/fendo.2023.1105602

**Published:** 2023-05-12

**Authors:** Jayna N. Mistry, Sanna Silvennoinen, Farasat Zaman, Lars Sävendahl, Katia Mariniello, Charlotte Hall, Sasha R. Howard, Leo Dunkel, Ulla Sankilampi, Leonardo Guasti

**Affiliations:** ^1^ Centre for Endocrinology, William Harvey Research Institute, Barts and the London Faculty of Medicine and Dentistry, Queen Mary University of London, London, United Kingdom; ^2^ Department of Pediatrics, Kuopio University Hospital and University of Eastern Finland, Kuopio, Finland; ^3^ Department of Women’s and Children’s Health, Karolinska Institutet and Karolinska University, Solna, Sweden

**Keywords:** GH signaling, GH resistance, FGF21, growth plate, preterm infants

## Abstract

**Background:**

Fibroblast growth factor 21 (FGF21) is an essential metabolic regulator that adapts to changes in nutritional status. Severe childhood undernutrition induces elevated FGF21 levels, contributing to growth hormone (GH) resistance and subsequent linear growth attenuation potentially through a direct action on chondrocytes.

**Methods:**

In this study, we assessed expression of the components of both GH and FGF21 pathways in rare and unique human growth plates obtained from children. Moreover, we investigated the mechanistic interplay of FGF21 on GH receptor (GHR) signaling in a heterologous system.

**Results:**

Chronic FGF21 exposure increased GH-induced GHR turnover and SOCS2 expression, leading to the inhibition of STAT5 phosphorylation and IGF-1 expression. The clinical significance of FGF21 signaling through GH receptors was tested in nutritionally driven growth failure seen in very preterm (VPT) infants right after birth. VPT infants display an immediate linear growth failure after birth followed by growth catch-up. Consistent with the *in vitro* model data, we show that circulating FGF21 levels were elevated during deflection in linear growth compared to catch-up growth and were inversely correlated with the length velocity and circulating IGF1 levels.

**Conclusions:**

This study further supports a central role of FGF21 in GH resistance and linear growth failure and suggests a direct action on the growth plate.

## 1 Introduction

Fibroblast growth factor 21 (FGF21), a member of the FGF19 subfamily of FGFs, is a key regulator in the metabolic adaptations to fasting, inducing gluconeogenesis, fatty acid oxidation and ketogenesis (1–3). FGF21 lacks the heparin binding domain allowing it to diffuse away from its tissue of synthesis and function as an endocrine factor (4, 5). FGF21 signals *via* FGFR1 isoform IIIC with the assistance of co-receptor β-Klotho (4, 6). Recent studies have identified a novel role of FGF21 as a potential candidate in developing GH resistance and growth failure with underlying chronic conditions (7, 8). Infants born very pre-term (VPT) (< 32 weeks) in intensive care are susceptible to undernutrition, causing poor linear growth and/or weight gain (9, 10). We previously highlighted a significant inverse association between serum FGF21 levels during the first 5 weeks of life and growth in length, but not for weight. In addition, we showed that FGF21 inhibits GH-induced pSTAT5 activity and *IGF-1* expression whilst stimulating *SOCS2* levels in human primary chondrocytes/chondroblasts established from ribs (1). Similarly, other studies showed serum FGF21 levels to be negatively associated with linear growth (11, 12), further emphasizing an inverse correlation with growth rates in infancy.

This study aimed to provide insight into the mechanistic interplay of FGF21 on GH receptor (GHR) and downstream signaling events involving the JAK/STAT cascade, directly linked with linear growth. Furthermore, the association between FGF21 levels and growth attenuation was evaluated to assess FGF21 levels during linear growth deflection and catch-up growth in a large cohort of VPT infants.

## 2 Materials and methods

### 2.1 Patients

A total of 64 VPT infants born before 32 gestational weeks (41 males, 64.1%) were recruited during the first week of life in the PreBaby study on metabolism and growth at the Kuopio University Hospital neonatal intensive care unit. The infants showed typical morbidity associated with prematurity (**Table 1**). However, they all survived until discharge. VPT infants were monitored regularly for their weight and recumbent length from birth (in-patient) to final growth follow-up (out-patient) at the mean age of 119.8 postmenstrual (PM) weeks (corresponding to around 18 months from the term-equivalent age of 40 PM weeks). The growth data were converted into Standard Deviation Score (SDS) using the contemporary population-based references (14).

**Table 1:** Clinical characteristics of the 64 VPT infants (41 males, 64.1%).

Multiple gestation (twins) n/%	27/42.2	
Bronchopulmonary dysplasia n/%	12/18.8	
Sepsis n/%	12/18.8	
Necrotizing enterocolitis n/%	2/3.1	
Severe (grade III-IV) IVH n/%	2/3.1	
Retinopathy of prematurity n/%	4/6.3	
	Mean	Range
At birth		
Gestational age, week	28.5	23.4 – 31.9
Weight, kg	1.14	500 – 1880
Weight, SD^a^	-0.49	-3.76 – 2.36
Length, cm	36.78	29.0 – 44.0
Length, SD^a^	-0.41	-4.70 – 4.10
At nadir		
Postmenstrual age for weight, week	33.6	28.0 – 40.0
Weight, kg	1.57	660 – 2630
Weight, SD^a^	-2.60	-4.88 – -0.49
Postmenstrual age for length, week	34.3	29.6 – 40.4
Length, cm	40.83	32.4 – 50.0
Length, SD^a^	-2.74	-6.05 – -0.43
Final growth follow-up visit		
Postmenstrual age, week	119.8	36.9 – 184.6
Weight, kg	10.45	2.81 – 15.60
Weight, SD^b^	-0.83	-4.49 – 1.87
Length, cm	80.72	46.80 – 98.20
Length, SD^b^	-0.70	-4.51 – 1.40

a Birth weight and length and weight and length at nadir were converted to SDS using the population-based birth size reference (13).

b Weight and length at the final growth follow-up visit were converted to SDS using the contemporary population based growth reference (13).

Peripheral venous or arterial samples were obtained at the age of 1, 3, 5, 7 and 9 weeks (in-patient) and at two follow-up visits after discharge (out-patient). Serum samples were prepared by centrifugation after blood collection, separated into aliquots and stored at -80˚C until analyzed.

Serum FGF21 concentrations were measured by human FGF21 ELISA kit (BioVendor, detection range 30 to 1920 pg/ml) using the manufacturer’s instruction. Serum IGF-1 concentration was measured by human IGF-1 ELISA kit (Mediagnost GmbH, detection range 2 to 50 ng/ml).

The growth pattern of VPT infants was evaluated during two distinct growth phases; 1) period of steady decrease in length/weight SDS (Growth deflection) and 2) period of steady increase in length/weight SDS (catch-up growth) differentiated by the point of nadir (the lowest point of length/weight SDS). The sampling of hormonal levels (FGF21 and IGF-1) was evaluated at the obtained timepoints, separated by the point of nadir to reflect the mean hormonal levels during growth deflection and catch-up growth.

### 2.2 Cells

HEK-293 were grown in DMEM high-glucose (4500mg/L, GIBCO) supplemented with 10% fetal-bovine serum (FBS, GIBCO) and 1% penicillin/streptomycin (P/S, Sigma) at 37°C in a humidified incubator with 5% CO_2_. Cells stably expressing GHR (HEK-293 hGHR) were generated by transfecting pCMV6-AC-Myc-DDK human GHR plasmid (Origene) into HEK-293 cells. Neomycin (500µg/ml; Sigma) was used as a positive selection marker.

### 2.3 PCR and quantitative RT-PCR

RNA extraction from human rib cartilage has been previously described (1). RNA from cells was obtained using RNeasy Mini Kit. 1μg of RNA was used to generate cDNAs: 1μg of random hexamers (New England Biolabs) was added to RNA samples to make a total volume of 15μl with Rnase/Dnase free water. The preparation was incubated at 70°C for 5 minutes for RNA denaturation (Veriti 96 well thermos cycler, Applied Biosystems). A master-mix made of 2μl of (10x) Moloney Murine Leukemia virus (M-MLV) reaction buffer (New England Biolabs), 1μl (10mM) deoxyribonucleotide triphosphate (dNTPs) (New England Biolabs), 1μl M-MLV RT (New England Biolabs) and 1μl Ribonuclease inhibitor (RNAsin) (New England Biolabs) was prepared per reaction sample. 5μl was added to the RNA sample following the initial incubation stage. Samples were further incubated at 25°C for 10 minutes, 42°C for 90 minutes and 70°C for 15 minutes for 1 cycle. cDNA was stored at -20°C.

PCR to detect *GHR*, *FGF21*, *FGFR1*, isoform *–IIIC*, *β-KLOTHO* and *GAPDH* was performed in a GS1 thermocycler (G-storm). Each reaction was prepared using 0.15µl (5U/µl) *Taq* polymerase (New England Biolabs), 2.5µl (10x) Standard *Taq* buffer (New England Biolabs), 0.5µl (200µM) of each dNTP, 1µl cDNA, 0.5µl (0.5µM) of specific primers and 20.35µl RNase/DNase free water.

Quantitative RT-PCR (RT-qPCR) reactions were prepared using 1µl of cDNA (~25ng), 0.5µl of each specific primer (0.5µM), 3µl dH_2_O and 5µl of SYBR green (QIAGEN) in Mx3000 thermocycler (Stratagene). Data was evaluated using MxPro software (Stratagene).

*GAPDH* was used as a house-keeping gene in PCR and RT-qPCR. Primers and cycle/amplification conditions are reported in **Supplementary Tables 1**, **2**.

The relative cycle threshold method (15) was used for the normalization and quantification of RT-qPCR data. Data are expressed as fold change relative to *GAPDH*.

10x cell ranger raw data (matrix, features and barcodes) was downloaded for P19 control mice from GEO Accession viewer (GSE162033). Data was imported to R (v. 4.2.2) using Seurat (v4.3.0).

Cells were filtered so that those with unique feature counts over 6000 or less than 200 and those with >10% mitochondrial counts were removed.

### 2.4 Single cell sequencing analysis

10x cell ranger raw data (matrix, features and barcodes) was downloaded for postnatal day 19 mice from GEO Accession viewer (GSE162033) (16). Data was imported to R (v. 4.2.2) using Seurat (v4.3.0). Cells were filtered so that those with unique feature counts over 6000 or less than 200 and those with >10% mitochondrial counts were removed. The global-scaling normalization technique “LogNormalize” was used to normalize the UMI count matrix. Seurat package “ScaleData” function was used to remove the unwanted sources of variation. The dimensionality of the dataset was determined using Seurat packages JackStraw and ElbowPlot. PC15 was decided the correct cut off to use going forward. Clusters were identified using ‘Find Neighbours’ and ‘FindClusters’ function. RunUMAP was used to create the UMAPs.

To annotate the clusters, differentially expressed features were identified by looking at the markers differing between clusters using the function “FindAllMarkers”. Plots were generated using Seurat functions, FeaturePlot and ggplot2.

### 2.5 Cell treatments

To assess glycosylation, HEK-293 hGHR cells (3 x 10^5^ cells per well in 6-well plates) were grown to reach ~90% confluency. Media was discarded and cells were washed with PBS before cell lysis with 20mM sodium phosphate (Sigma) pH 7.5, 0.1% sodium dodecyl sulfate (Sigma), 0.75% Nonidet P-40 (Sigma), 50mM β-mercaptoethanol (Sigma) and protease tablet inhibitor cocktail (Sigma), for 20 minutes in ice. Cells were then centrifuged at 13,000rpm for 10 minutes at 4°C, and the supernatant was collected. 20µl of cell lysate was treated, with or without N-glycosidase F (New England Biolabs) overnight at 37°C. The enzymatic reaction was stopped by 2x Laemmli buffer (Sigma) and processed for Western blotting.

To assess GH and FGF21 responsiveness, HEK-293 hGHR cells (3 x 10^5^ cells per well in 6-well plates) were grown to reach 80% confluency. Cells were serum starved overnight and treated in the absence or presence of Cycloheximide (CHX) (100µg/ml; Sigma), recombinant GH (0.5µg/ml; Life Technologies) or recombinant FGF21 (5µg/ml; VWR) for 1 – 8 hours and then processed for GHR expression using Western blotting.

HEK-293 hGHR cells were grown at 3 x 10^5^ cells per well in 6-well plates. The following day, cells were serum starved and treated with or without recombinant FGF21 (5µg/ml) overnight before being challenged with recombinant GH (0.5µg/ml) for 10 and 30 minutes for the assessment of pSTAT5(Ty694) by Western blotting or 8 and 16 hours for the evaluation of *SOCS2* and *IGF-1* mRNA expression *via* RT-qPCR.

### 2.6 Cell proliferation

96-well plates were coated with 100µl per well of collagen (collagen I, Rat tail (1µl; GIBCO), acetic acid (0.67µl; Fisher Scientific), 50µl dH_2_O) for 1 hour at 23°C. Collagen was aspirated and wells were washed twice with PBS and air-dried for 2 hours. HEK-293 hGHR cells were plated at 5 x 10^3^ cells per well and grown to reach 50% confluency before being serum starved in the absence or presence of recombinant FGF21 (5µg/ml) overnight. Cells were then challenged with recombinant GH (0.5µg/ml) for 96 hours. Media was removed and cells were treated with 100µl cell-counting reagent kit-8 (10:100 dilution in serum free media; Sigma) incubated at 37°C for 1 hour. The absorbance was measured at 450nm with a Microplate Reader (Bio-Rad)

### 2.7 Immunohistochemistry and RNAScope

Human liver paraffin sections were obtained from Generon. Human growth plate biopsies were obtained from pediatric patients undergoing epiphyseal surgery to arrest longitudinal bone growth due to constitutional tall stature or leg length difference and cultured *ex-vivo* as previously described (17). Sections were then incubated at 60°C for 40 minutes, deparaffinized in xylene (twice for 10 minutes), and then hydrated in graded alcohol (ethanol) baths; 99% ethanol (twice for 5 minutes), 95% ethanol (10 minutes), 70% ethanol (5 minutes) and dH_2_O (10 minutes). Antigen retrieval was performed in citric acid (0.01M) and sodium citrate (0.1M) in dH_2_O at 80°C for 20 minutes. After, sections were washed with PBS twice and with PBS 0.01% Tween followed by incubation with 3% goat serum for 1 hour and overnight incubation with primary antibodies at 4°C (**Supplementary Table 3**). Sections were washed in PBS 0.01% Tween (five times), followed by incubation with secondary antibody; goat anti-rabbit IgG (Vector Laboratories) or goat anti-mouse IgG (Santa-Cruz Biotechnology) at 1:200 dilution in 1% BSA in PBS for 1 hour at 23°C. After washing in PBS 0.01% Tween for 20 minutes, peroxidase activity was detected under a microscope using DAB-kit (Vector Laboratories). The reaction was stopped by rinsing the section with dH_2_O and counterstained with Alcian blue. Dehydration of sections was performed in graded alcohol baths; dH_2_O, 70% ethanol, 95% ethanol, 99% ethanol and xylene each for 5 minutes and mounted with Pertex (Histolab products AB).

Human liver sections were processed for human FGF21 RNAScope (**Supplementary Table 5**) using the 2.5 HD Assay- BROWN Kit (ACD) according to the manufacturer’s instructions.

### 2.8 Immunoprecipitation

HEK-293 hGHR cells (1 x 10^5^ cells per/ml in T75cm^2^ flasks) were grown to reach 70% confluency. Cells were serum starved in the absence or presence of recombinant FGF21 (5µg/ml) overnight before being challenged with recombinant GH (0.5µg/ml) for 16 hours. Cells were lysed in 1ml RIPA buffer (Sigma) supplemented with protease inhibitor cocktail (Sigma) and kept on ice for 20 minutes following centrifugation at 13,000rpm for 10 minutes. Supernatant was collected and reacted with protein A/G plus agarose beads (30µl; Santa-Cruz Biotechnology) on a rotor at 4°C for 1 hour. Samples were centrifuged at 2500rpm for 5 minutes and the sample was transferred to a new Eppendorf tube followed by antibody incubation; normal rabbit IgG or anti-GHR B-10 on a rotor at 4°C overnight. 30µl of protein A/G plus agarose beads were added to each sample and placed on a rotor at 4°C for 2 hours. The samples were centrifuged at 2500rpm for 5 minutes and the supernatant was discarded. The cell pellet was washed with RIPA buffer and twice with PBS centrifuging at 2500rpm for 5 minutes between washes. 80µl of 2X Laemmli buffer was added, and the sample boiled at 95°C for 5 minutes followed by centrifugation at 13,300rpm for 1 minute and transferred to a new Eppendorf tube and processed for Western blotting.

### 2.9 Western blotting

Cell lysates obtained from HEK-293 hGHR cells were size-separated on a 10% SDS gel, against the PageRuler, Plus Prestained protein ladder (ThermoFisher Scientific) and blotted onto a nitrocellulose membrane (GE Health care Life sciences). Membranes were incubated with blocking buffer, 5% non-fat dry milk (Asda) in PBS containing 0.1% Tween-20 for 1 hour at 23°C followed by incubation with primary antibody (**Supplementary Table 4**) prepared in blocking buffer overnight at 4°C.

Membranes were washed with PBS 0.1% Tween-20 (three times, 15-minute intervals) and then incubated with secondary antibody with goat anti-mouse IRDye680/800 and goat anti-rabbit IRDye680/800, dilution 1:10,000 (LI-COR). Immunoblots were scanned using the Odyssey Infrared Imaging System (LI-COR)

### 2.10 Study approval

This study was approved by the Ethics Committee of the Pohjois-Savo Health Care District, Finland. Informed consent was obtained from both parents of all study participants.

The local ethical committee, (Karolinska Institutet Research Ethics Committee North at the Karolinska University Hospital, Stockholm, Sweden) approved the collection of human growth plate biopsies. Informed consent was obtained from each subject and their parents, which was also documented in the original hospital records.

### 2.11 Statistical analyses

Clinical data evaluation was performed in SPSS software (version 24.0). FGF21 and IGF-1 serum concentrations were not normally distributed when tested using the Kolmogorov-Smirnov test and therefore the values were first log transformed to achieve normality of residuals. Log transformed FGF21/IGF-1 levels at weeks 1, 3, 5, 7 and 9 postnatally (in-patient) and weeks 1 and 2 (out-patient) after birth were separated individually for each patient and averaged to reflect mean levels during growth deflection and catch-up growth. Statistical tests to evaluate hormonal levels during deflection and catch-up growth included bivariate correlation and paried *t*-test analysis.

*In vitro* data were evaluated by One-way ANOVA, Dunnett’s *post hoc* test, Two-way ANOVA, Bonferroni post *t*-test or Non-linear regression one phase decay using GraphPad Software version 5. Each experiment was performed a minimum of 3 times. Data are expressed as mean ± standard error of the mean (SEM). Statistical significance was defined as *p* values <0.05.

## 3 Results

### 3.1 GHR, FGF21 and FGF21 receptor complex are expressed in the human growth plate

**Figure 1:**
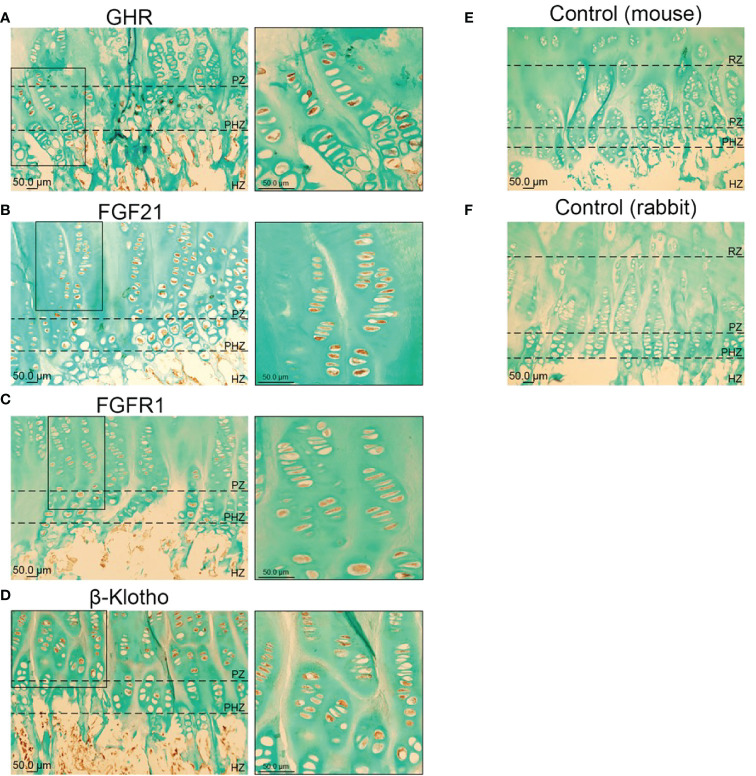
Immunohistochemical localization of GHR **(A)**, FGF21 **(B)**, FGFR1 **(C)** and β-KLOTHO **(D)** in male human growth plate tissue (tibia) in late puberty; mean age 12.3 years (*n*=6). Negative control: Human growth plate tissue incubated with secondary antibody, goat anti-mouse **(E)** and goat anti-rabbit **(F)**, (*n*=6).

Previous data, including *ex-vivo* cultures of human chondroblasts with a pre-hypertrophic/proliferative phenotype (1) and *in vivo* animal studies (2) alluded that FGF21 could act directly at the level of growth plate; however, whether components of the FGF21 pathway are expressed in the growth plate is not known. Immunohistochemical analysis showed that expression of GHR, FGF21, FGFR1 and β-Klotho was highly localized in the proliferative and pre-hypertrophic zones (**Figures 1A–F**; **Supplementary Figure 1**), suggestive of a potential functional crosstalk between GH and FGF21 pathways in these cells, regulating longitudinal growth. In contrasts with immunohistochemistry, analysis of single cell sequencing datasets of mouse growth plates (18) indicated extremely low counts of *Fgf21* compared to *GhR* or *Fgfr1*, suggesting either differences in expression between mice and humans or a potential cross-reactivity of anti-FGF21 antibody in the human growth plate samples. *GhR* or *Fgfr1* were enriched in clusters enriched with chondrocyte lineage progenitors and growth plate chondrocytes, albeit proliferative and pre-hypertrophic zones could not be safely identified in the single cell clusters (**Supplementary Figure 2**).

### 3.2 Development of an *in vitro* model to investigate GH/FGF21 crosstalk

**Figure 2:**
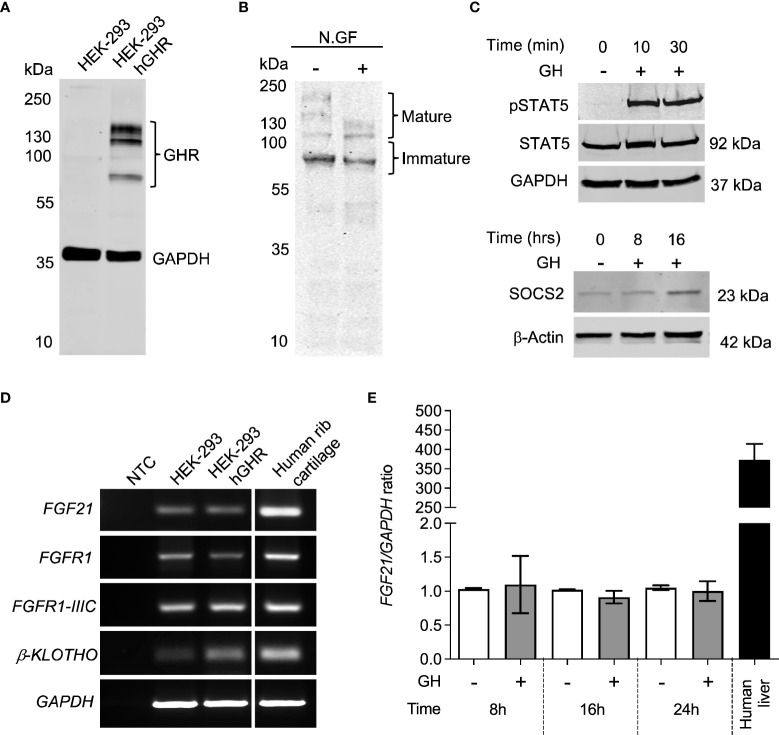
HEK-293 exogenously expressing GHR are responsive to GH. **(A)** Western blot analysis of GHR expression in HEK-293 (control) and HEK-293hGHR with GHR antibody. The molecular weight (kDa) of protein standard is reported on the left. **(B)** Protein extracts obtained from HEK-293 hGHR cells were incubated with or without N-glycosidase F (N.GF) and immunoblotted with GHR antibody. The band at ~140 kDa represent the glycosylated mature GHR while the lower m.w. bands represent immature GHR in the ER and Golgi. **(C)** (upper panel) HEK-293hGHR cells were serum starved overnight and incubated with or without recombinant human GH (0.5µg/ml) for 10 or 30 minutes. Whole cell lysates were subjected to western blot analysis of STAT5 (92kDa) and pSTAT5(Tyr694) protein expression; (lower panel) HEK-293hGHR cells were serum starved overnight and challenged with recombinant human GH (0.5µg/ml) for 8 and 16 hours. Whole cell lysates were subjected to western blot analysis of SOCS2 (23kDa) protein expression. **(D)** PCR analysis of *FGF21, FGFR1*, *isoform -IIIC, β-KLOTHO*, and *GAPDH* in HEK-293 and HEK-293hGHR stable line cells; negative controls (NTC) were PCR samples where cDNA was omitted. Human rib cartilage was used as a positive control. **(E)** HEK-293 hGHR cells were serum starved overnight and challenged with GH (0.5 µg/ml) for 8, 16 and 24 hours before analysis of endogenous *FGF21* expression by RT-qPCR. All values are relative to untreated cells at 8 hours. n=3 applies to all panels.

We next aimed at further assessing the functional crosstalk between FGF21 and GH pathways by establishing a GH- and FGF21-responsive *in vitro* model. HEK-293 cells stably expressing human GHR (HEK-293hGHR) displayed multiple GHR bands in Western Blot experiments, representing both ER-resident and plasmamembrane/mature GHR (**Figures 2A, B**), as described by others (12). Heterologous GHR was functional as recombinant human GH induced rapid STAT5 phosphorylation and increased negative regulator SOCS2 expression (**Figure 2C**). HEK-293hGHR endogenously expressed FGF21 receptor complex; *FGFR1*, isoform *FGFR1-IIIC* and *β-KLOTHO*, indicating their potential responsiveness to FGF21 (**Figure 2D**). HEK-293hGHR also expressed *FGF21*, although at significant lower levels compared to human liver; interestingly, GH had no effect on the endogenous expression of *FGF21* suggesting that FGF21 induced GH-resistance may partially be driven by chronic hepatic FGF21 levels (**Figure 2E**).

### 3.3 FGF21 enhances GH-mediated GHR turnover

**Figure 3:**
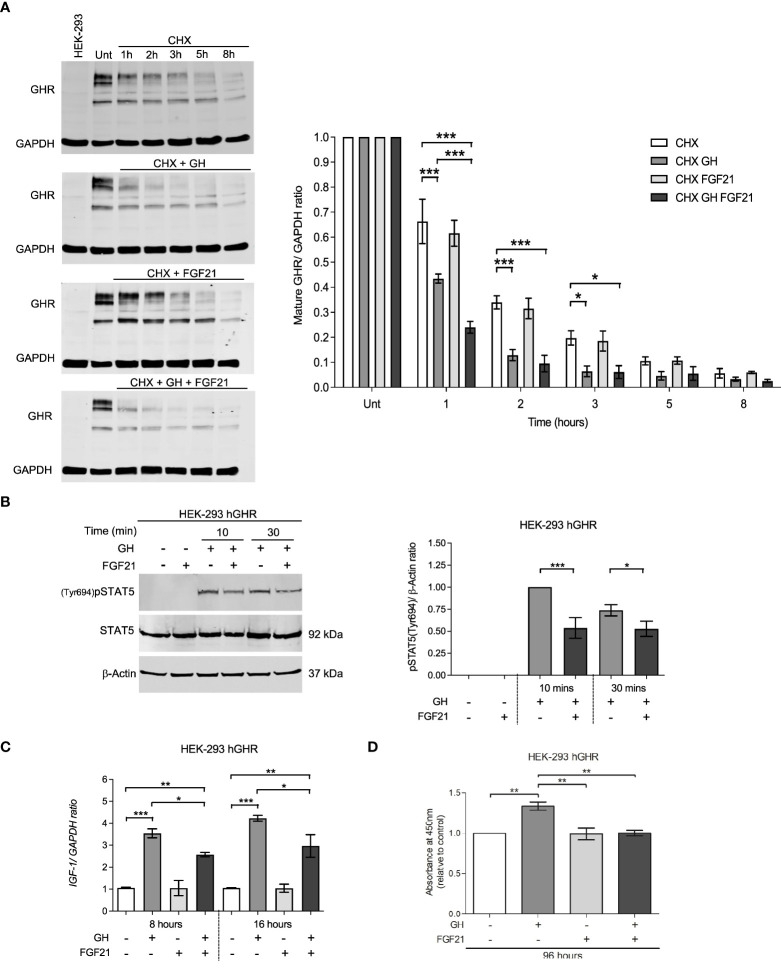
FGF21 decrease GHR half-life and inhibits downstream GHR signaling events. **(A)** HEK-293hGHR cells were serum starved overnight and incubated in the absence or presence of CHX (100 µg/ml), GH (0.5 µg/ml) or FGF21 (5 µg/ml) at the indicated timepoints up to 8 hours before analysis of GHR by Western blot; Unt: Untreated at 0 hours; HEK-293, non-transfected cells. The right panel indicates the ratio of mature GHR *vs.* untreated. 0 hours normalized to housekeeping GAPDH. (right bottom panel) Quantification of the rate (hours) of mature GHR half-life, expressed as % of time at 0 hours, *n*=4. **(B)** HEK-293hGHR cells were serum starved and treated in the absence or presence of FGF21 (5 µg/ml) overnight and then challenged with GH (0.5 µg/ml) for 10 to 30 minutes before analysis of pSTAT5(Tyr694) by Western blot (left panel). The right panel indicates the ratio of pSTAT5(Tyr694) *vs.* total protein, normalized to GH treatment alone at 10 minutes, *n*=5. **(C)** HEK-293hGHR cells were serum starved with or without FGF21 (5 µg/ml) overnight and then challenged with GH (0.5 µg/ml) for 8 or 16 hours before analysis of *IGF-1* expression by RT-qPCR, *n*=3. **(D)** HEK-293hGHR cells were serum starved with or without FGF21 (5 µg/ml) overnight and then challenged with GH (0.5 µg/ml) for 96 hours before analysis of cell proliferation, *n*=3. *p<0.05; **p<0.01; ***p<0.001.

GH action depends on the availability of GHR on the cell surface and its baseline turnover can be accelerated by GH itself *via* proteolysis and ligand-induced endocytosis (19). We tested the hypothesis that FGF21 might affect the amount of GHR on the cell surface by modulating GHR turnover. HEK-293hGHR were treated with cycloheximide (CHX) to block protein translation, and mature GHR protein levels were assessed over a period of 8 hours by Western Blot. CHX treated cells challenged with GH resulted in a significant reduction in GHR half-life at 1 hour (*p*<0.0001), 2 hours (*p*<0.0001) and 3 hours (*p*=0.136) compared to CHX treated cells only (**Figure 3A**, left panel). The rate of mature GHR degradation was significantly reduced in CHX + GH treated cells compared to CHX treated only from 1.355 to 0.7741 hours (*p*=0.0007) (**Figure 3A**, right panel). This is in keeping with data reported by other investigators (19). No significant differences were detected in mature GHR half-life when cells were treated with CHX + FGF21 compared to CHX treatment alone. Interestingly, a greater shift and rapid reduction of GHR half-life was observed when cells were concomitantly treated with FGF21 and GH with the expression of mature GHR almost abolished by 5 to 8 hours (**Figure 3A**). This difference in mature GHR expression was most significant at the 1-hour time-point (*p*=0.0003) (**Figure 3A**, right panel). The rate of mature GHR degradation was significantly reduced in FGF21 + GH, treated cells compared to GH alone, from 0.7741 to 0.4518 hours (*p*=0.0015) (**Figure 3A**, right panel). These results suggest that FGF21 enhances GH-mediated GHR turnover and degradation *in vitro*.

We next investigated whether FGF21 could affect GHR-mediated signal transduction. FGF21 inhibited GH-induced STAT5 phosphorylation at 10 and 30 minutes compared to GH treatment alone (**Figure 3B**). This also led to a reduction in *IGF-1* mRNA expression at both the timepoints analyzed (8 and 16 hours) compared to GH treatment alone (**Figure 3C**). Furthermore, treatment with FGF21 and GH significantly inhibited cell proliferation compared to GH treatment alone (**Figure 3D**).

**Figure 4:**
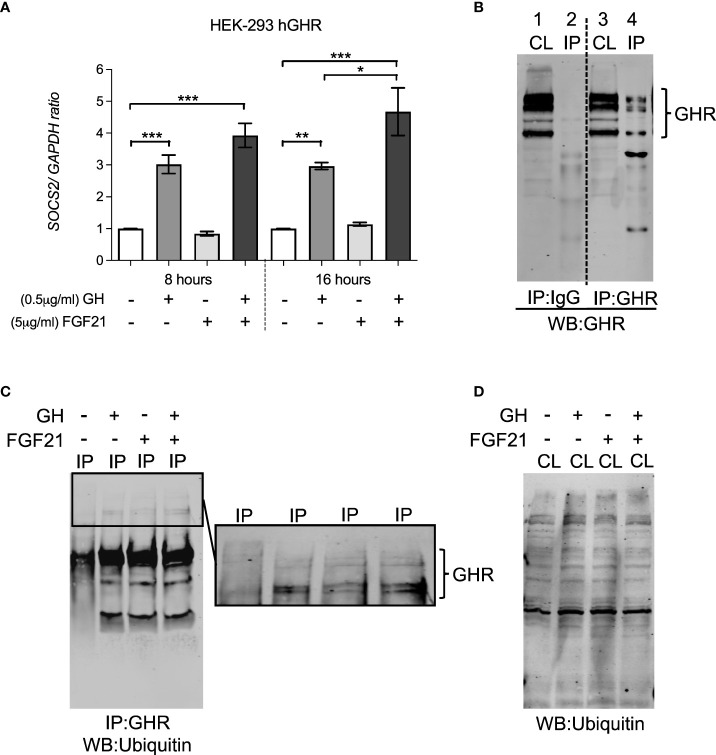
FGF21 increased GH-induced *SOCS2* expression but had no effect on the ubiquitination of GHR. **(A)** HEK-293hGHR cells were serum starved in the absence or presence of FGF21 (5 µg/ml) overnight and then challenged with GH (0.5 µg/ml) for 8 or 16 hours before analysis of *SOCS2* by RT-PCR (left panel) **(B)** Anti GHR can immunoprecipitate GHR in HEK-293hGHR cells: cells were immunoprecipitation with antiGHR antibodies or control IgG. Cell lysates and immunoprecipitated were developed with antiGHR antibodies CL, cell lysate; IP, immunoprecipitated sample; HC, heavy chain IgG; LC, light chain IgG. Negative control; IgG. **(C)** Western blot of GHR ubiquitination in immunoprecipitated samples after treatment with GH with or without FGF21. **(D)** Western blot of ubiquitin in whole cell lysates (*n*=3). *p<0.05; **p<0.01; ***p<0.001.

SOCS2 affects GHR function either *via* binding to phosphorylated tyrosine residues on GHR, blocking downstream signaling events, or by regulating cellular GHR levels by facilitating GHR ubiquitination and proteasomal dependent degradation (20, 21). In HEK-293hGHR, FGF21 was able to potentiate GH-induced upregulation of *SOCS2* expression (**Figure 4A**), however, chronic FGF21 had no effect on GH-induced ubiquitination of GHR (**Figures 4B–D**). Taken together, these results suggest that FGF21 increased GH-mediated GHR degradation resulting in attenuation of downstream signaling (pSTAT5), culminating in a reduction of *IGF1* expression. This mechanism could be mediated by FGF21-dependent upregulation of SOCS2, leading to GHR degradation.

### 3.4 Linear growth trends in VPT infants and FGF21 levels

**Figure 5:**
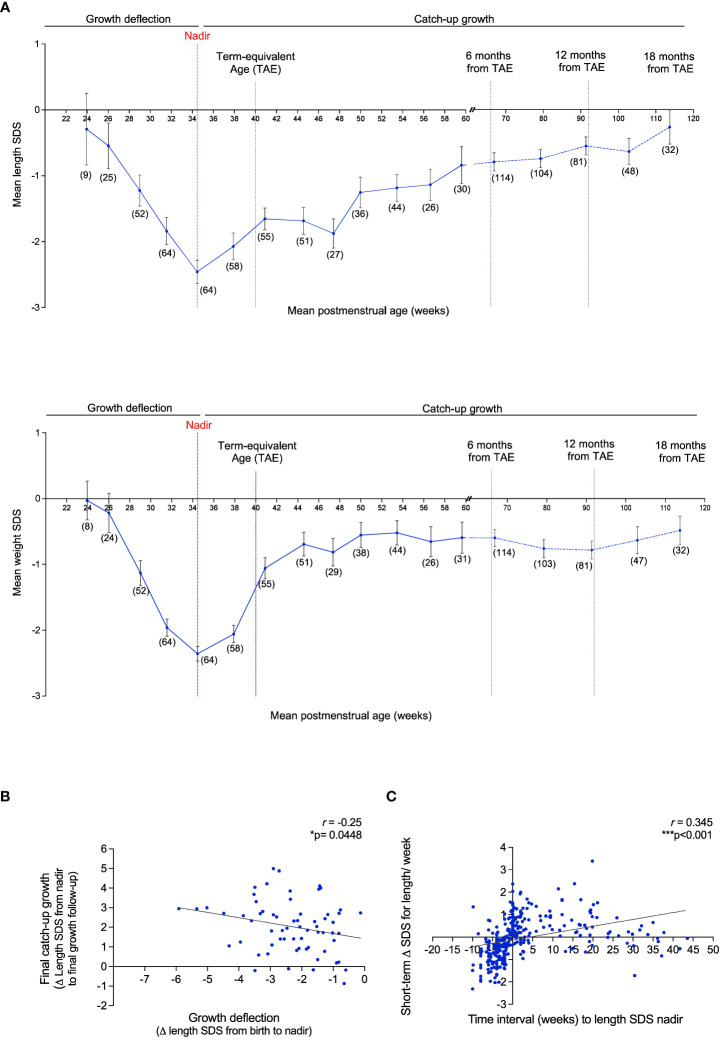
Growth pattern of VPT infants born <32 gestational weeks display an immediate postnatal growth failure in length and weight followed by a catch-up growth after nadir. **(A)**: Mean length SDS (top panel) and weight SDS (bottom panel) at the average of 3 week intervals from birth to 61 postmenstrual (PM) weeks (corresponding to 4 months of corrected age from the term-equivalent age of 40 PM weeks), and at 12 week intervals thereafter, until 119.8 PM weeks (corresponding to 18 months of corrected age). Observations per a sub-group at certain PM age (weeks) is reported as *n*=(x). Error bars represent the mean ± SEM. Nadir: Lowest length/weight SDS score. **(B)**: Association of the magnitude of growth deflection (ΔSDS for length from birth to nadir) and total catch-up growth (ΔSDS for length from nadir to final growth follow-up). **(C)**: Assessment of ΔSDS for length/week (length assessed at the age of 1, 3, 5, 7 and 9 weeks during hospitalization) and time interval (weeks) to the length SDS nadir. 511 Pearson’s R correlation (r) and p values were obtained from Bivariate correlation analyses.

VPT infants displayed a relatively uniform growth pattern, consisting of a poor linear growth rate immediately after birth, as evident by a rapid decrease in the mean length/weight SDS (growth deflection). This was followed by a period of catch-up growth, observed by an increase in the mean length/weight SDS after nadir (lowest point of length/weight SDS) (**Figure 5A**). At nadir the average length SDS was -2.74 SDS, which was seen at an average age of 34.3 PM weeks. The average weight SDS at nadir was -2.60 SDS, which was seen at an average age of 33.6 PM weeks. The magnitude of deflection (ΔSDS for length from birth to nadir) was significantly negatively correlated with total catch-up growth (ΔSDS for length from nadir to final growth follow-up) (*r*= -0.25, *p*= 0.045) (**Figure 5B**), such that infants who experienced a greater decline in SDS during the deflection stage exhibited a greater SDS increase during the catch-up phase. Evaluation of length SDS (assessed at the age of 1, 3, 5, 7 and 9 weeks during hospitalization) in comparison to catch-up growth at two follow-up visits after discharge (out-patient) demonstrated that VPT with a greater degree of growth deflection were more susceptible to a more negative short-term Δ in length SDS, whilst faster catch-up growth was reflective of a more positive short-term Δ in length (r= 0.35, p< 0.001) (**Figure 5C**).

**Figure 6:**
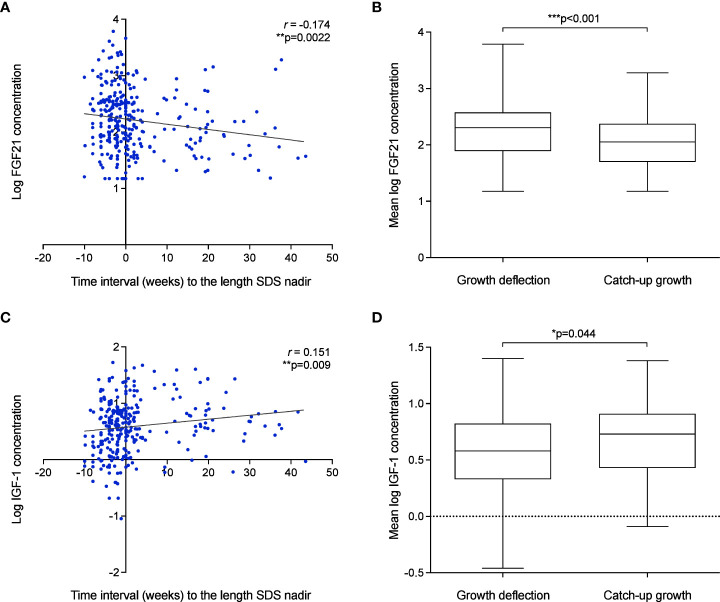
Growth failure after birth in VPT infants is associated with elevated FGF21 and low IGF-1 levels. **(A–D)**: The association of FGF21 **(A)** and IGF-1 **(C)** concentration and time interval (weeks) to the length SDS nadir. Pearson’s R correlation (*r*) and *p* values were obtained from Bivariate correlation analyses. Assessment of mean FGF21 **(B)** and IGF-1 **(D)** concentration during deflection and catch-up growth phase. *p* values were obtained from Paired *t*-test analysis.

Individual serum FGF21 and IGF-1 levels were evaluated at weeks 1, 3, 5, 7 and 9 (in-patient) and at two follow-up visits after discharge (out-patient) to assess the correlation between hormonal levels with time interval (weeks) to the length SDS nadir. A weak but significant negative correlation was observed between LogFGF21 levels and time interval (weeks) to the length SDS nadir (*r* = -0.17, *p*=0.002) (**Figure 6A**). Thus, FGF21 levels were higher during deflection (mean = 483.35 pg/ml, *SD* = 581.32) than during catch-up (mean = 223.04 pg/ml, *SD* = 211.50; *p*<0.001) (**Figure 6B**). This correlation was not seen without logarithmic correction of FGF21 concentrations (**Supplementary Figures 4A, C**). Inversely, there was positive correlation between IGF-1 levels and time interval (weeks) to the length SDS nadir (*r* = 0.15, *p*=0.009) (**Figure 6C** and **Supplementary Figures 3B, D**), and consistently, IGF-1 levels were higher during catch-up (mean 5.63ng/ml, *SD* = 5.46) than during deflection (mean 7.82ng/ml, *SD* = 7.13; *p*=0.044) (**Figure 6D**).

## 4 Discussion

The clinical evaluation on VPT infants undertaken in this study has broadened our understanding of the role of elevated circulating FGF21 levels in the development of GH resistance and subsequent poor linear growth outcomes. It is well described that premature birth poses long-term health risks, particularly associated with growth failure which is further magnified by underlying chronic conditions i.e., undernutrition (9, 10, 22–25). However, the molecular signals leading to GH insensitivity remain ill-defined.

Recruitment of VPT infants as part of a clinical evaluation has thus offered an applicable model to investigate the unknown mechanisms associated with childhood growth failure. Our cohort of VPT infants showed a uniform growth trend demonstrated by an immediate growth deficit after birth, followed by catch-up growth after the point of nadir. This unique linear growth pattern in pre-term infants has been widely reported across some clinical studies (26, 27). Interestingly, an independent evaluation of deflection and catch-up growth revealed an upregulation in FGF21 levels during deflection compared to catch-up growth, which was inversely correlated with IGF-1 levels. This offers evidence to suggest that upregulation in FGF21 levels as seen during growth deflection may lead to growth failure in VPT infants potentially *via* mechanisms of GH resistance evaluated in our *in vitro* observations. The period of catch-up growth appears to be marked by the opposite – alleviation of FGF21 mediated GH resistance.

The potential functional cross talk between GH and FGF21 signaling was studied in the human growth plate and in a cell model responsive to both hormones. GHR and FGF21/FGF21 receptor complexes were both expressed in chondrocytes of the proliferative and pre-hypertrophic zone; their expression in the resting zone should be investigated in biopsies contain an intact resting zone, which was not possible in our samples. The proliferative, pre-hypertrophic and hypertrophic growth plate zones play an essential role in chondrocyte proliferation and maturation where GHR signaling occurs to promote longitudinal bone growth (28–30). The inability to reliably detect endogenous GHR with antibodies in many experimental settings prompted us to generate a line heterologously expressing a detectable GHR. We showed that HEK-293hGHR responded to recombinant GH whilst endogenously expressing the FGF21 receptor complex. This allowed us to assess, for the first time, the effects of recombinant GH and FGF21 on the turnover of GHR and the activity of GHR-activated pathways such as pSTAT5, SOCS2, IGF1 expression and GHR ubiquitination. Growth plates (**Figure 1**), rib cartilage and HEK-293hGHR (**Figure 2**) expressed FGF21 suggesting a potential paracrine/autocrine mechanism of action; despite this, the amount of FGF21 expressed in the HEK-293hGHR was significantly lower compared to the liver. Moreover, GH had no effect on the endogenous expression of FGF21 and can therefore be excluded as a potential co-factor in regulating FGF21 levels.

Intriguingly, chronic FGF21 was seen to increase GH-induced GHR turnover. This may be due to a functional interaction between the GH and FGF21 pathways with unknown molecular events linking the two. The action of FGF21 on GHR degradation/internalization led to the abrogation of GHR signaling events. This was demonstrated through the rapid reduction in signaling components including STAT5 phosphorylation and IGF-1 expression. Thus, the inhibitory effect of chronic FGF21 levels on early (pSTAT5) and late (IGF-1) downstream events of the GHR signaling cascade, reveals FGF21s’ ability to develop GH resistance, highlighting its role in linear growth attenuation.

A second independent action of chronic FGF21 was confirmed on GH-induced SOCS2 upregulation. We speculate that an increase in the negative feedback regulator SOCS2, acts as an additive effect on the role of chronic FGF21 in GH-induced GHR degradation by further prohibiting the activation of GHR signaling to induce a state of GH resistance. It is well described that SOCS2 exerts its actions *via* two main mechanisms. 1) *via* binding to phosphorylated tyrosines on GHR resulting in blocking the association of positive signaling regulators (JAK2 and STAT5b activation), 2) *via* regulating cellular GHR levels by direct ubiquitination in a proteasomal dependent manner (21, 31). Interestingly, FGF21 did not increase GH-induced ubiquitination of cell surface GHR, despite our observations of FGF21s’ actions on increased GHR turnover/degradation and SOCS2 expression. An inability to detect subtle albeit biologically relevant changes in ubiquitination in the western blot setting may explain why FGF21 was not seen to induce GHR ubiquitination. Further investigation is required to explore alternative mechanisms of GHR ubiquitination potentiated by chronic FGF21 e.g. *via* ubiquitin independent proteasomal degradation.

Overall, a thorough evaluation of the mechanistic role of chronic FGF21 on GHR signaling has provided further insights into the direct actions of FGF21 in the development of GH resistance. These findings suggest two main mechanisms of FGF21 in GH insensitivity and subsequent growth failure. 1) Chronic FGF21 increases GHR turnover, reducing the activation of GH-induced STAT5 phosphorylation and IGF-1 expression. 2) Chronic FGF21 upregulates GH-induced SOCS2 expression, suppressing GHR activation and downstream signaling events. As we employed a suitable, but inherently imperfect *in vitro* model, appropriate validation in either *ex vivo* primary cultures of growth plate/organoids or *in vivo* models would be needed to fully substantiate the proposed mechanisms.

The clinical evaluation of growth trends in VPT infants further provided evidence to suggest that elevated FGF21 levels as seen during growth deflection may drive GH insensitivity and stunted growth. The regulation of FGF21 expression associated with GH resistance, however, warrants further experimental evaluation. Several recent investigations describe the interplay of macronutrient (carbohydrates, fatty acids, and proteins) availability on the endogenous expression of hepatic and circulating FGF21 levels (32–34). Our data did not distinguish whether the observed declines in FGF21 and increase in IGF-1 are driven by the change in nutritional state or by increasing age. We did not have access to detailed nutritional data for this cohort; assessment of the nutritional intake in another cohort of VPT infants may highlight a potential co-factor associated with upregulating FGF21 expression.

This study has opened new avenues for an opportunity to consider more tailored treatment strategies in children with debilitating chronic conditions. Novel treatment approaches will aid to enhance therapeutic management, having a significant cost saving effect for failed GH treatment in the healthcare system. Ultimately, advancement in clinical outcomes associated with GH resistance will improve the overall quality of life of infants in both the short and long-term.

## Data availability statement

The raw data supporting the conclusions of this article will be made available by the authors, without undue reservation.

## Ethics statement

The studies involving human participants were reviewed and approved by Pohjois-Savo Health Care District, Finland. Written informed consent to participate in this study was provided by the participants’ legal guardian/next of kin.

## Author contributions

LG, LD, US, and SH: conception and design of the study, supervision, acquisition of data, analysis and interpretation of data, writing the article, and approval of final version. JM and SS: conception and design of the study, performed experiments, analysis and interpretation of data, and writing the article. FZ and LS: acquisition of data, analysis and interpretation of data. KM: performed RNAScope experiments. CH: performed single cell sequencing analysis. All authors contributed to the article and approved the submitted version.
