# Supplementary material for: The crosstalk between FGF21 and GH leads to weakened GH receptor signaling and IGF1 expression and is associated with growth failure in very preterm infants
Source: Front Endocrinol (Lausanne). 2023 May 12;14:1105602. doi: 10.3389/fendo.2023.1105602 (PMC10213667; doi:10.3389/fendo.2023.1105602)
Supplement: Supplementary file 1 [file DataSheet_1.pdf]

Supplementary data

Tables

Name Forward (F) Reverse (R)	Species Human (H) Mouse (M)	Forward primer sequence in 5'-3' orientation	Reverse primer sequence in 5'-3' orientation
**PCR**			
GHR	H	GGTATGGATCTCTGGCAGCTG	GAGGGCAATGGGTGGATCTG
FGF21	H	ACCTGGAGATCAGGGAGGA	AGTGGAGCGATCCATACAGG
FGFR1	H	GAAGTTCAAATGCCCTTCCA	CCAGCTGGTATGTGTGGTTG
FGFR-IIIC	H	ACCACCGACAAAGAGATGGA	GCAGAGTGATGGGAGAGTCC
β-KLOTHO	H	GCTCTCAAAGCCCACATAC	GCAGCATAACGATAGAGGCC
GAPDH	H	GAAGGTGAAGGTCGGAGT	GAAGATGGTGATGGGATTTC

Table 1 – PCR thermocycler reaction cycle conditions and primer sequence. Thermocycler RT-PCR reaction cycle conditions for *(GHR, FGF21, FGFR1 β-KLOTHO and GAPDH)*: polymerase activation for 3 minutes at 95°C, followed by 35 cycles of denaturation for 3 seconds at 95°C, 30 seconds annealing temperature (see table below for specific temperatures) and extension for 20 seconds 72°C. Extra-extension was for 3 minutes at 72°C. PCR reaction cycle conditions for *(FGFR1 IIIC* isoform*)*: polymerase activation 5 minutes 95°C, followed by 35 cycles denaturation for 1 minute at 95°C, annealing for 1 minute at 65°C and extension for 1 minute at 72°C). Extra-extension was for 10 minutes at 72°C.

Name Forward (F) Reverse (R)	Species Human (H) Mouse (M)	Forward primer sequence in 5'-3' orientation	Reverse primer sequence in 5'-3' orientation
**RT-qPCR**			
FGF21	H	QuantiTect Primer Assay (Qiagen).	
GAPDH	H		
SOCS2	H	GGTCGAGGCGATCAGTG	TCCTTGAAGTCAGTGCGAA
IGF-1	H	TGGTGGATGCTCTTCAGTTC	GACAGAGCGAGCTGACTTG
GHR	H	GTGATGCTTTTTCTGGAAGTGA	TCAGGGCATTCTTTCCATTC
FGFR1	H	GAAGTTCAAATGCCCTTCCA	CCAGCTGGTATGTGTGGTTG
FGFR1-IIIC	H	ACCACCGACAAAGAGATGGA	GCAGAGTGATGGGAGAGTCC
β-KLOTHO	H	GCTCTCAAAGCCCACATAC	GCAGCATAACGATAGAGGC

Table 2 – RT-qPCR cycle conditions and primer sequences. Mx3000 Thermocycler reaction conditions: polymerase activation at 95˚C for 15 minutes, followed by 40 cycles of denaturation at 95˚C for 15 seconds, annealing at 60˚C for 30 seconds and extension at 72˚C for 30 seconds.

Antibody	Source (code), species	Dilution
GHR (B-10)	Santa Cruz Biotechnology (sc-137185), mouse	1:25
FGF21	Abcam (ab66564), rabbit	1:100
FGFR1	Abcam (ab63601), rabbit	1:50
β-Klotho	Abcam (ab106794), rabbit	1:300

Table 3 – Primary antibody list for immunohistochemistry.

Antibody	Source (code), species	Dilution
GHR (B-10)	Santa Cruz Biotechnology (sc-137185), mouse	1:500
Stat5 (C-17)	Santa Cruz Biotechnology (sc-835), rabbit	1:500
Phospho-Stat5 (Tyr 694) (C11C5)	Cell Signaling Technology (9359), rabbit	1:500
SOCS2	Abcam (ab3692), rabbit	1:500
Ubiquitin (Ubi-1)	Abcam (ab7254), mouse	1:500
GAPDH (G9)	Santa Cruz Biotechnology (sc-365062), mouse	1:10,000
Beta Actin (AC-15)	Abcam (ab6276), mouse	1:10,000

Table 4 – Primary antibody list for western blot analysis.

Gene	Source	Probe target region
Human FGF21	ADC	13-842

Table 5 – Probe used for Single molecule RNAScope

Supplementary Figures

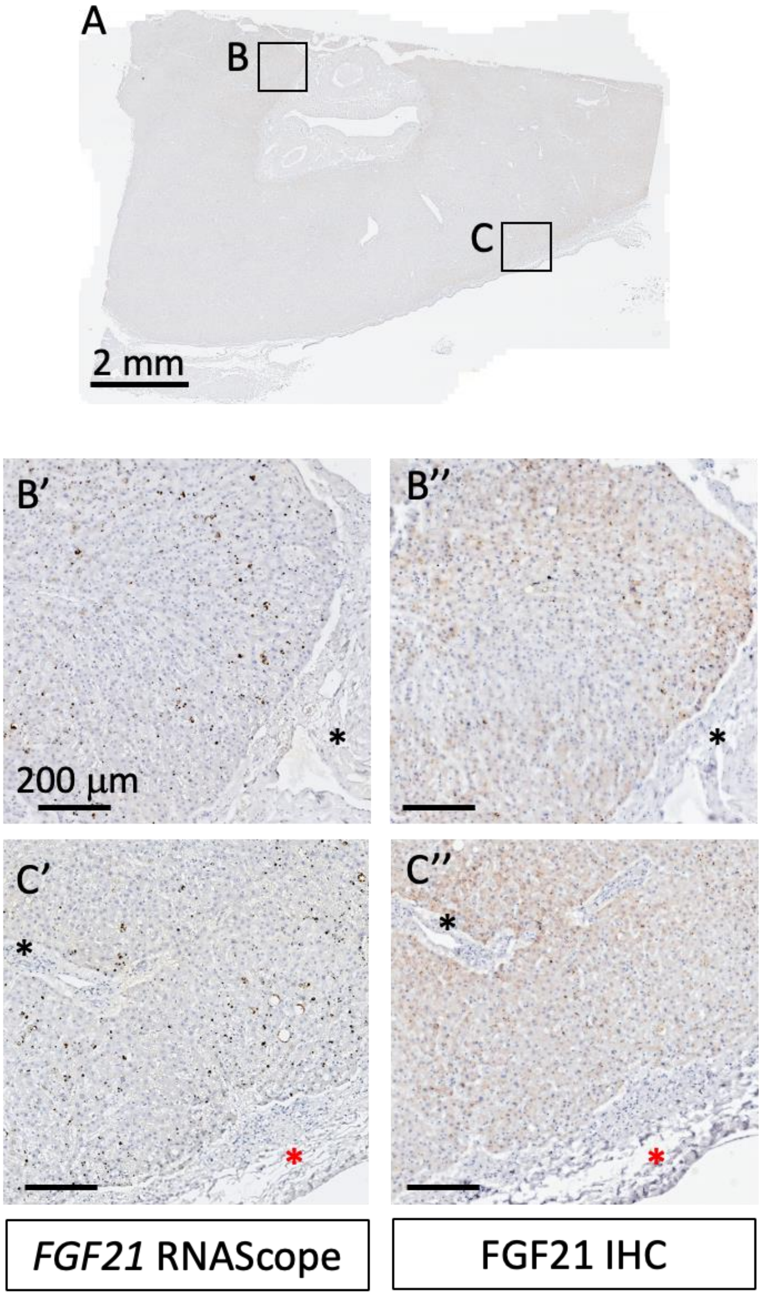

Supplementary Figure 1. Testing anti-FGF21 antibody ab66564. Consecutive sections of formalin-fixed, paraffin embedded human liver were processed for single molecule RNAScope (A, B' and C') and immunohistochemistry using FGF21 antibodies (B'' and C''). Note the presence of staining in the liver parenchyma at the mRNA and protein level, and absence of specific signal in vascular structures (black asterisks in B' and B'') and in the fibrous capsule (C' and C'').

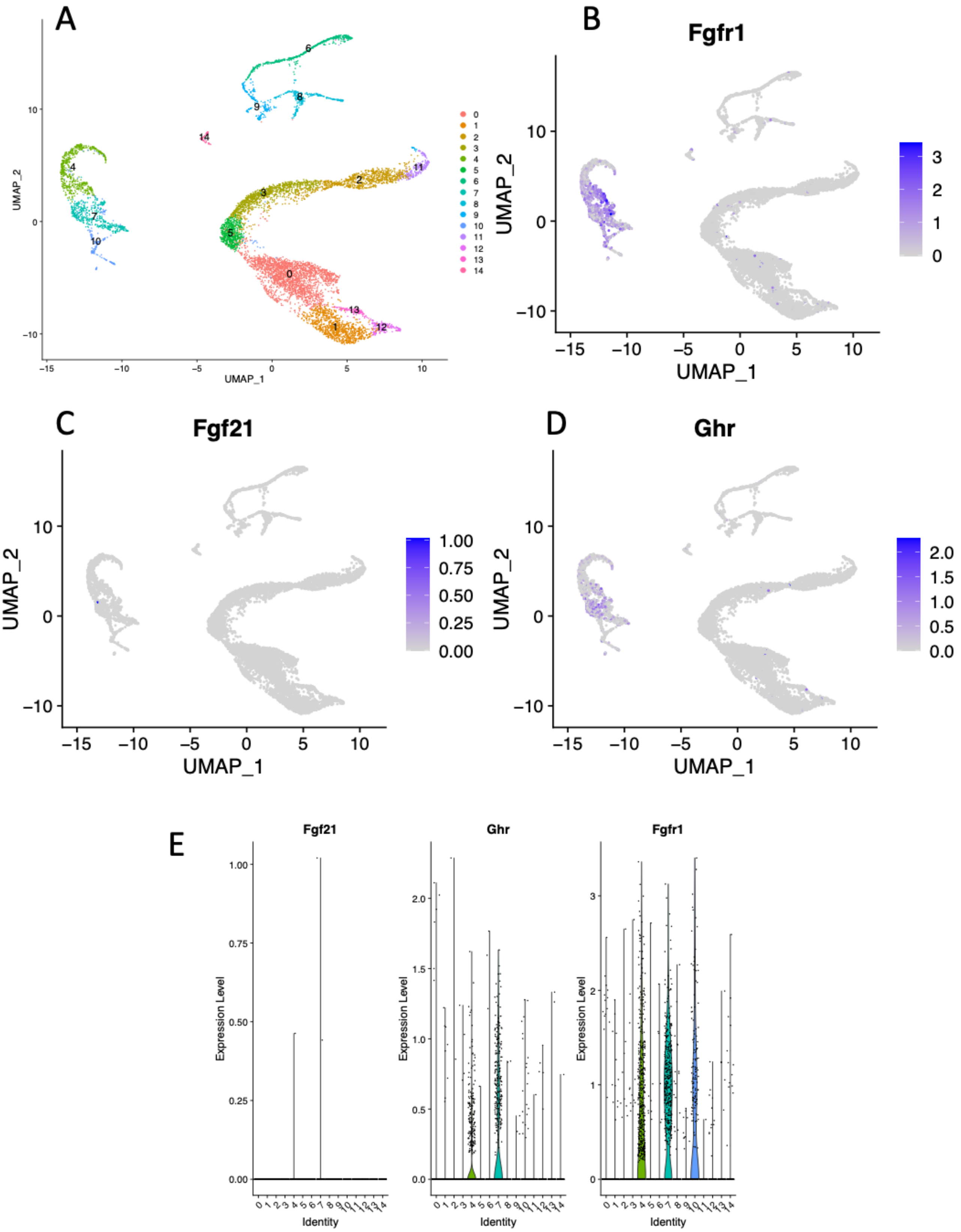
A
B
Fgfr1
C
Fgf21
D
Ghr
E
Fgf21
Ghr
Fgfr1
UMAP_1
UMAP_2
Expression Level
Identity

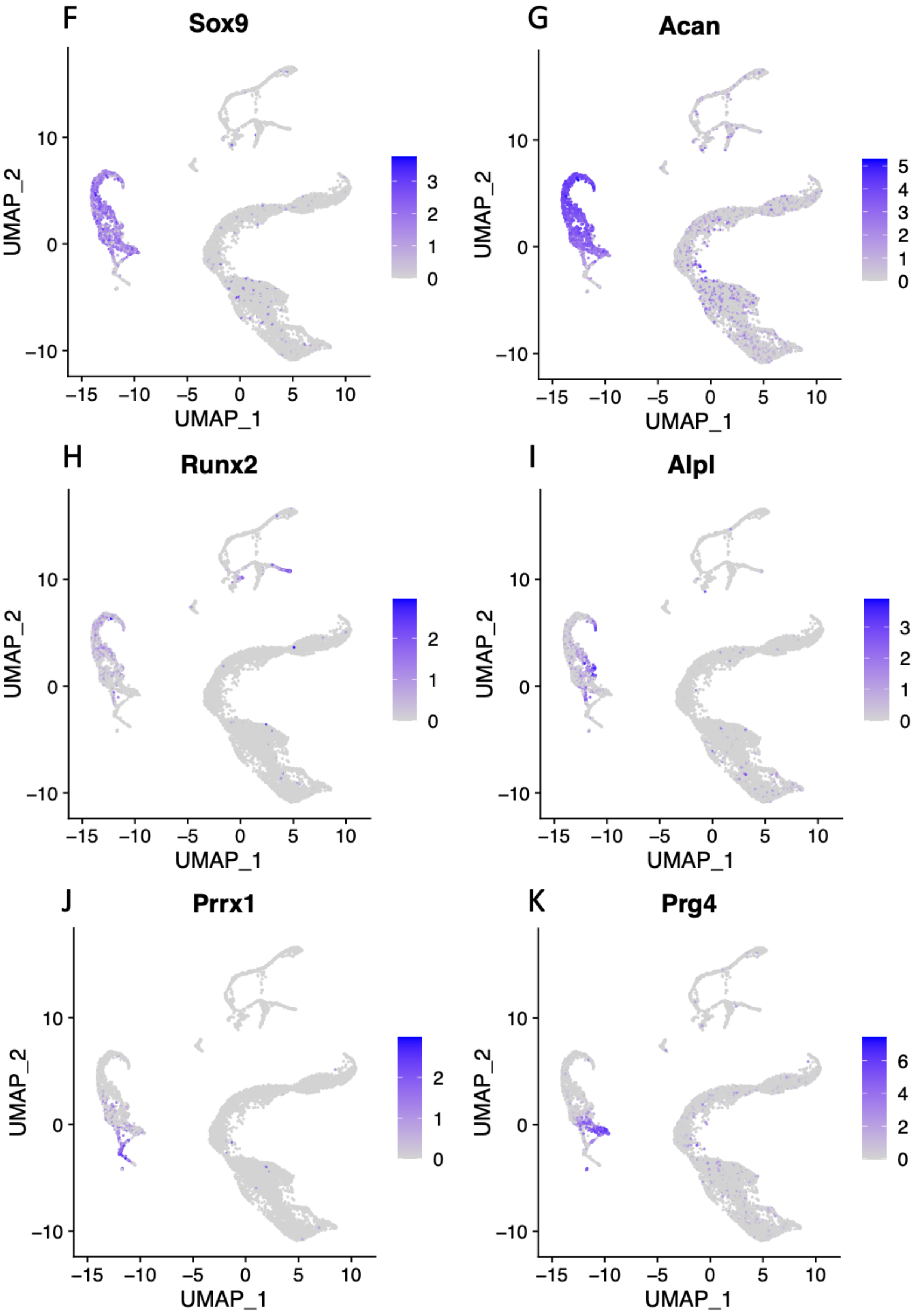
F
Sox9
G
Acan
H
Runx2
I
Alpl
J
Prrx1
K
Prg4
UMAP_1
UMAP_2

Supplementary Figure 2. A) Uniform Manifold Approximation and Projection (UMAP) plot of skeletal cell populations extracted from mouse femoral and tibial epiphyses of postnatal day 19. Cells were segregated into 14 clusters. B) UMAP plots relative to FGFR1 (B), FGF21 (C) and GHR (D), end violin plots of their expression levels (E). Significantly lower counts for FGF21 were detected compared to FGFR1 and GHR. Expression was preponderant in clusters 4, 7 (FGF21, GHR) and 4,7,10 (FGFR1).
F-K) UMAP plots relative of lineage specific markers: SRY-Box Transcription Factor 9 (Sox9, chondrocyte lineage progenitors, F), Aggrecan (Acan, Chondrocyte progenitors, G), RUNX Family Transcription Factor 2 (Runx2, skeletal progenitors, growth plate chondrocytes and osteoblasts, H), alkaline phosphatase, biomineralization associated (Alpl, growth plate chondrocytes and osteoblasts, I), Paired Related Homeobox 1 (Prrx1, skeletogenic mesenchymal cells, J), Proteoglycan 4 (Prg4, superficial articular chondrocytes, K).

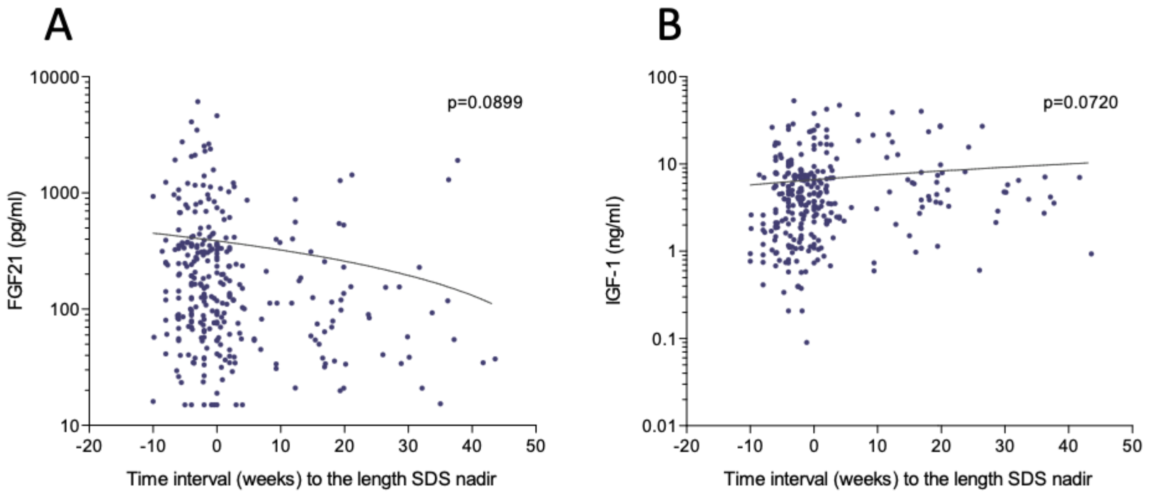

Supplementary Figure 3. The association of FGF21 (A) and IGF-1 (C) concentration and time interval (weeks) to the length SDS nadir, expressed as log of FGF21 serum levels.

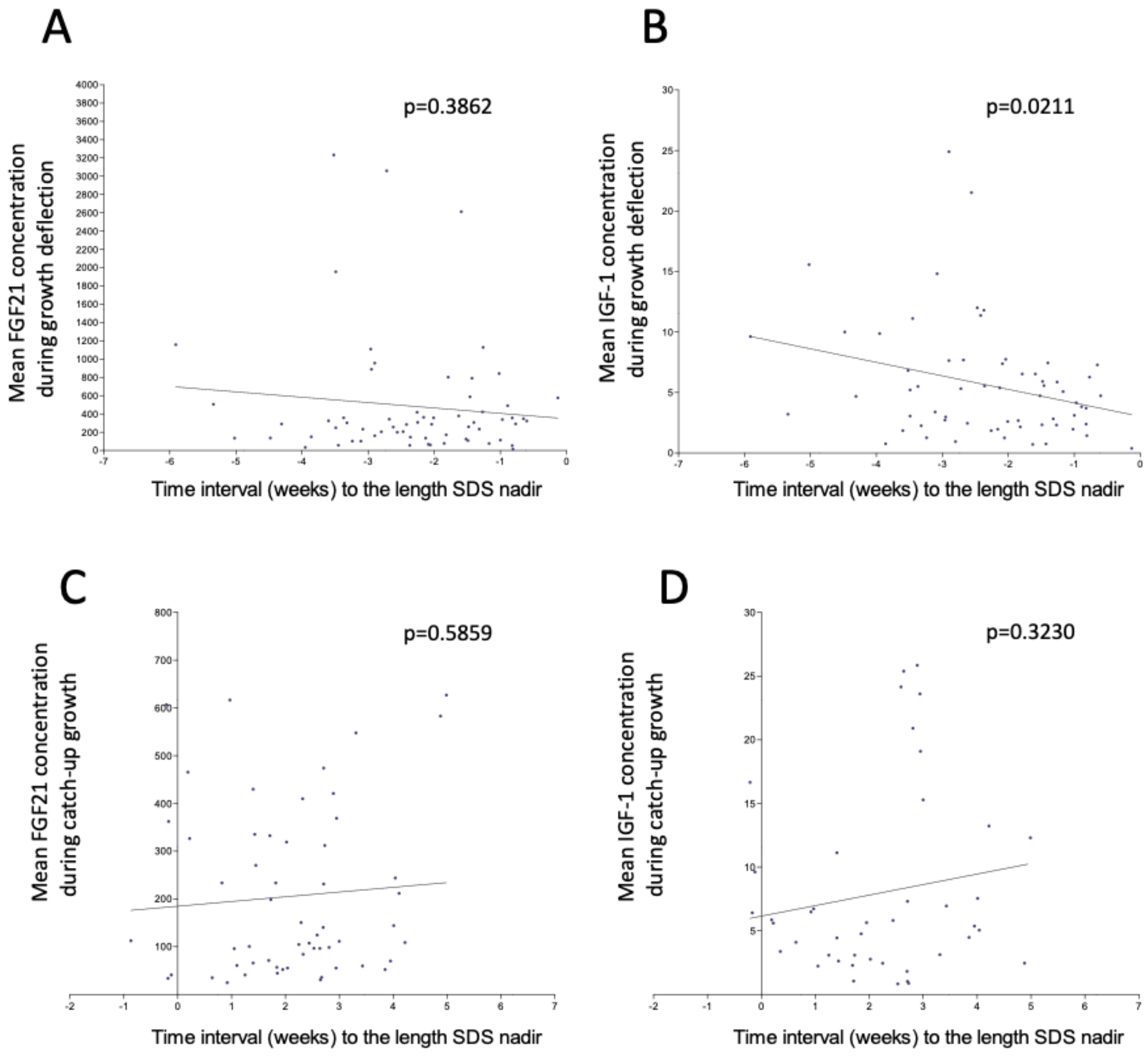

Supplementary Figure 4. Correlation between mean FGF21 (A), IGF1 (B) levels and time interval (weeks) to the length SDS nadir during growth deflection. Correlation between FGF21 (C) and IGF1 (D) levels and time interval (weeks) to the length SDS nadir during catch-up growth.
